# Outcome of Endoprosthesis used in Limb Salvage Surgery in a Malaysian Orthopaedic Oncology Centre

**DOI:** 10.5704/MOJ.2403.008

**Published:** 2024-03

**Authors:** YH Ng, YC Chai, N Mazli, NF Jaafar, S Ibrahim

**Affiliations:** 1 Department of Orthopaedic, Hospital Sultan Ismail, Johor Bahru, Malaysia; 2 General Psychiatry Division, Hospital Permai Johor Bahru, Johor Bahru, Malaysia; 3 Department of Orthopaedics and Traumatology, Universiti Kebangsaan Malaysia, Kuala Lumpur, Malaysia

**Keywords:** primary bone tumour, secondary bone metastasis, endoprosthesis reconstruction

## Abstract

**Introduction:**

To describe the duration of survival among bone tumour patients with endoprosthesis reconstruction and to determine frequency of implant failure, revision of surgery, and amputation after endoprosthesis reconstruction.

**Materials and methods:**

A retrospective cross-sectional review of all patients with either primary bone tumour or secondary bone metastases treated with en bloc resection and endoprosthesis reconstruction from January 2008 to December 2020.

**Results:**

A total of 35 failures were recorded among the 27 (48.2%) patients with endoprostheses. Some of the patients suffered from one to three types of modes of failure on different timelines during the course of the disease. Up to eight patients suffered from more than one type of failure throughout the course of the disease. Out of all modes of failure, local recurrence (type 5 failure) was the most common, accounting for 25.0% of all failure cases. Four patients (7.1%) eventually underwent amputation, which were either due to infection (2 patients) or disease progression causing local recurrence (2 patients).

**Conclusion:**

The overall result of endoprosthesis reconstruction performed in our centre was compatible with other centres around the world. Moreover, limb salvage surgery should be performed carefully in a selected patient group to maximise the benefits of surgery.

## Introduction

The Malaysia National Cancer Registry report 2012-2016 stated that the lifetime risk of males getting cancer is 1 in 10 and the ratio among females is 1 in 9^1^. Sarcoma is rare and only accounts for 1% of all malignancies. Sarcomas, on the other hand, are the second most common type of solid tumour in children^2^. Limb salvage surgery is the treatment of choice in more than 90% of patients with primary bone tumours^1-3^. It involved resection of the diseased bone and skeletal reconstruction with either biological (autograft, allograft) or non-biological (endoprosthesis) or combination (allograft prosthetic composite). Limb salvage surgery, rather than amputation, became possible as a result of advances in imaging technique, chemotherapy regime and modern prosthetic design^3-5^.

There are up to 280,000 new cases of secondary bone metastasis diagnosed per year in developed countries, and the number is expected to be on the rise as life expectancy increases with advancements in the healthcare system^6^. Metastatic bone is weakened and necessitates skeletal stabiliation with an allograft, orthopaedic implant or endoprosthesis that should last the patient’s lifetime^7^. Endoprosthesis reconstruction is indicated in most of the cases involving meta-epiphyseal metastasis. Endoprosthesis provide quick intra-operative reconstruction of the bone defect after resection and can provide durable and immediate skeletal stability to allow for post-operative rehabilitation.

The orthopaedic department of Hospital Sultan Ismail (HSI), Johor Bahru is the tertiary referral centre for sarcoma cases and skeletal-related events secondary of metastasis tumours in the southern region of peninsular Malaysia since 2008. Limb salvage surgery is one of the most important surgeries performed by the orthopaedic oncology team in this hospital. Both biological and non-biological reconstruction have been performed in this centre. Biologic reconstruction is preferred in younger primary bone tumour patients and performed together with contralateral epiphysiodesis in order to achieve as little limb length discrepancy as possible. However, in older children that near or attained skeletal maturity, endoprosthesis reconstruction is preferred and this group of patients constitute the majority of our patients. Endoprosthesis reconstruction for patients with either primary or secondary malignancy of the bones only were included in this study. Herein, we sought to review the outcome of the endoprosthesis surgery that had been performed in our centre for the past 13 years. The study’s objectives were to describe the survival of endoprosthesis reconstruction and to determine the frequency of implant failure, revision of surgery. and amputation after endoprosthesis reconstruction.

## Materials and Methods

This is a retrospective cross-sectional study that was carried out from November 2022 to February 2023. It involved a retrospective review of patients’ databases in the orthopaedic department of HSI. All the primary bone tumour and secondary bone metastasis patients treated with en bloc resection and endoprosthesis reconstruction at HSI from January 2008 to December 2020 were included, with a minimal follow-up of at least 24 months.

The extracted data included the demographic data of patients, histological diagnoses, complications after the surgeries, and dates of patients’ mortality. The outcomes of the study related to the survival of the endoprosthesis are according to the definition suggested by Henderson *et al*^8^. Five modes of failure were described, which included soft tissue failures (Type 1), aseptic loosening (Type 2), structural failures (Type 3), infection (Type 4), and tumour progression (Type 5)^8^. Revision of an endoprosthesis due to any of these 5 types of failure or amputation was recorded.

All patients received chemotherapy or radiotherapy as per hospital protocol. All patients were screened for superficial skin infection before deciding for an operation. Chlorhexidine soap bath was instructed a day before the operation date. Standard skin preparations with chlorhexidine soap bath and povidone iodine were performed prior to surgical field draping. Prophylactic antibiotics (intravenous cefuroxime, 1500mg) were given half an hour before the skin incision. Intravenous cefuroxime 750mg continued to serve eight hourly post-operation for the next day. Duration of limb salvage surgery ranges from three to seven hours. All endoprosthesis used was cemented. Any leaking of bone cemented around the implant was removed meticulously. Haemostasis was secured and the wound drain inserted in all cases to prevent post-operative hematoma collection. Routine medial gastrocnemius flap was done in all cases of proximal tibia endoprosthesis. All wounds were closed in layers without tension. Wound inspection and dressing post-operatively were performed under sterile conditions.

Patients with humerus endoprosthesis were put on slingshot arm brace for one month for soft tissue recovery. Patients who had endoprosthesis of the proximal femur were keep on hip abduction pillow for two days. Do and don’t on hip arthroplasty education was taught to patients while in the ward. Patients with endoprosthesis reconstruction surgery around the knee will be put in a knee brace for four to six weeks. The brace was initially kept fully extended before gradually allowing flexion for patients with patella tendon reconstruction. Patients mobilised with partial weight bearing on post-operative day 3 and gradually increased to full weight bearing as tolerated for all patients with lower limb surgery. All patients were referred to a physiotherapist for intensive physiotherapy as part of their post-operative rehabilitation strategy.

Upon discharge from the ward, scheduled dressing at the polyclinic will be informed. The oncology team would inform regarding the surgery and to plan for subsequent treatment either for radiotherapy or chemotherapy accordingly. The first follow-up in the orthopaedic clinic would be two weeks after the initial surgery. Subsequently, monthly for three months, then every three months for one year, then every six months for two years then yearly follow-up. During each follow-up, physical examination would be carried out, and particular attention would be given to look for signs of inflammation, evidence of local recurrence and metastasis. Chest radiograph and radiograph of the operated site would be performed at least six-monthly for the first two years or when there is a new complaint. Blood parameter testing, computed tomography or magnetic resonance imaging scan would be carried out whenever it is indicated during follow-up. Those with evidence of implant failure will be documented and managed accordingly.

Data on the mortality of patients who underwent endoprostheses have been obtained via a default tracking system in the orthopaedic clinic at HSI since the establishment of the orthopaedic oncology service in 2008. If any patients did not show up for their follow-up appointment more than two months after the last defaulted clinic appointment, clinic staff would contact the patients or next-of-kin to schedule a new appointment. If the patient’s family informed clinic that he or she had died, the clinic staff would record the date of death in the patient’s clinical notes. Furthermore, all patients and their families were reminded to report their progress (include death) to the doctors in charge or clinic staff on a regular basis. Those who receive the information would document it in the patients’ clinical notes. In addition, data on patients’ mortality could be captured via hospitalisation notes. It is because some of the patients may have been admitted to the ward as a result of tumour complications, which eventually caused them to die in the hospital.

## Results

The basic demographic data about the patients showed in Table I. Between 2008 to 2020, 56 patients with musculoskeletal tumours, either primary or secondary, underwent endoprosthesis reconstructions. The ages range from 11 to 75, with a mean of 35.93±17.45. According to Table I, the majority of the patients were male (55.4%), with a higher proportion of Malay patients (60.7%). The most common bone that was affected by the tumour is the femur (66.1%), in which distal femur involvement accounted for up to 44.6% of the cases. Osteosarcoma was the most common pathology (46.4%). The giant cell tumour was the only non-malignant tumour that required an endoprosthesis in up to 19.6% of patients. Endoprosthesis reconstructions were performed on 8 patients (14.3%) with metastatic carcinoma to the long bones. Four out of 8 patients succumbed at the time of writing, and the mean time of survival was 27 months. 14% of the primary bone tumour cases were diagnosed with lung metastasis prior to the surgeries. After endoprosthesis surgery, approximately 32% of primary bone tumours developed metastasis; the mean time and median time to develop metastasis were 20.3 months and 11.5 months, respectively. A total of 26 patients (46.4%) succumbed, which included 22 out of 26 patients with primary bone tumours who developed metastases either pre-or post-operatively. The mean duration of survival was 25 months after the index surgery.

**Table I:** Basic demographic data of the patients with musculoskeletal tumours who underwent endoprosthesis reconstruction.

	Mean (SD)	Frequency (n)	%
Age	35.93 (17.45)		
Gender			
Male		31	55.4
Female		25	44.6
Race			
Malay		34	60.7
Chinese		15	26.8
Indian		5	8.9
Others		2	3.6
Pathology			
Osteosarcoma		26	46.4
Ewing sarcoma		4	7.1
Giant cell tumour		11	19.6
Bone metastasis		8	14.3
Other primary bone tumour		7	12.5
Metastasis			
Primary carcinoma with bone secondary metastasis		8	14.3
Sarcoma with metastasis (pre-operative)		8	14.3
Sarcoma with metastasis (post-operative)		18	32.1
No metastasis		22	39.3
Survival			
DOD		26	46.4
SWD		30	53.6
Site of tumour			
Whole femur		5	8.9
Proximal femur		12	21.4
Distal femur		17	30.4
Proximal tibia		12	21.4
Distal femur and proximal tibia		3	5.4
Humerus		7	12.5
Margin of surgery			
Clear margin		46	82.1
Margin involved		10	17.9
Tissue necrosis post chemotherapy in primary bone sarcoma			
≥90%		11	35.5
< 90%		20	64.5
Mortality		26	46.4

** total number of patients, n=56; DOD: dead of disease; SWD= survive with disease

The types of failure in relation to types of malignancy and sites of endoprosthesis showed in Table II. Collectively, 35 failures were recorded among 27 patients (48.2%) with limb salvage surgeries. If Type 5 failure is excluded, a total of 17 patients (30.4%) suffered from 4 other types of failure. In fact, Type 5 failure more related to the disease than the implant problem. Some of the patients suffered from one to 3 types of failure on different timelines in the course of the disease. There were eight patients who suffered from more than one type of failure throughout the course of the disease. Local recurrence (Type 5 failure) was the highest type of failure in comparison to all other modes of failure. Only one of the 14 patients with local recurrence was associated with secondary bone metastasis. The other 13 patients were: 9 patients with osteosarcoma or Ewing’s sarcoma; 2 patients with giant cell tumours; and the other 2 patients were related to primary bone sarcomas. Only one out of the 9 patients from the osteosarcoma and Ewing’s sarcoma groups showed a good chemotherapy response of 90% tumour necrosis, while the rest had a poor chemotherapy response, ranging from 0-85% tumour necrosis with a mean of 62%. Surgical margin involvement was noted in 2 cases of osteosarcoma among those with Type 5 failure. Structural failure (Type 3 failure) and infection (Type 4 failure) accounted for 9 cases each. Three patients (5.4%) suffered from Type 1 failure, and none of the patients suffered from Type 2 failure. Four patients (7.1%) eventually underwent amputation, with 2 due to infection and the other 2 due to disease progression causing local recurrence. Out of nine cases of structural failure, revision surgery was performed on six patients; two patients succumbed to the illness and the other patient did not wish for another operation due to personal reasons.

**Table II:** Types of failure in relation to types of malignancy and site of endoprosthesis.

Implant failure n=35	Type1 Soft tissue failure (n=3)	Type 2 Aseptic loosening (n=0)	Type 3 Structural failure (n=9)	Type 4 Infection (n=9)	Type 5 Tumour progression (n=14)
Pathology					
Osteosarcoma	3	0	6	6	8
Ewing sarcoma	0	0	1	0	1
Giant cell tumour	0	0	1	0	2
Bone metastasis	0	0	1	0	1
Other primary bone tumour	0	0	0	3	2
Total	3	0	9	9	14
Site of tumour					
Whole femur	0	0	1	1	3
Proximal femur	0	0	1	2	1
Distal femur	1	0	3	3	4
Proximal tibia		0	2	1	3
Distal femur and proximal tibia	1	0	1	1	2
humerus	1	0	1	1	1
Total	3	0	9	9	14

## Discussion

Limb salvage surgery is the first choice of treatment in most of the tumour cases. It consists of complete removal of tumour, reconstruction of the bone defect and soft tissue closure. The bone defect reconstruction is either biological or non-biological. The option of reconstruction is mainly based on tumour prognosis, skeletal maturity, remaining bone and soft tissue, and patient or family expectations^9,10^. Biological reconstruction can be either allograft or autograft. Autograft can be either recycling, vascularised or non-vascularised. Vascularised bone graft is the best option for biological reconstruction as it maintains the physiological blood supply of the graft hence its viability and healing ability at the graft-recipient junction^11^. However, the usage is limited by high technical demands of the operation and scarce supply of donors site^12^.

Non-biological reconstruction with endoprosthesis offers considerable advantages in terms of function outcome, appearance, and psychological acceptance^13^. Since the first modular endoprosthesis was introduced in 1980, it had largely replaced the custom-made implant^14^. It allows intra-operative assembly as required after the resection of the tumour. However, complications associated with the endoprosthesis, such as the various types of implant failure mentioned by Henderson *et al* had raised significant concern among operating surgeons^8^. Disease progression with local recurrence (Type 5 failure) is closely related to the aggressiveness of the malignancy rather than implant problems. Certain authors excluded Type 5 failure from their study and reported an overall implant failure rate after excluding Type 5 failure that ranged from 20-30%^14,15^. After excluding Type 5 failure, the failure rate in our centre was 30.4%, which is comparable to other reported results.

Advances in implant design, particularly the introduction of the rotating hinge system, reduced implant strain and reduced the incidence of the aseptic loosening (Type 2 failure) to the lowest of all failure types^16^. In our centre, none of the patients suffered from aseptic loosening, which is consistent with the conclusion drawn by other authors. Mechanical failure (Type 3 failure) and infection (Type 4 failure) were the leading causes that limited the survival of the endoprosthesis implant^13^. Structural failure accounts for 16.1% of failures among our patients. A constrained implant with a long lever arm at the implant-bone interface places high stress on the components of the endoprosthesis and might predispose to mechanical failure^13^. In view of most of our patients are also from the young age group that have high physical demand, this had put significant stress to the endoprosthesis that lead to structural failure.

The median time to develop infection (Type 4 failure) after endoprosthesis reconstruction was 24 months, and the risk of implant-related infection persisted throughout the life of the prosthesis at a mean rate of 1% per year^13^. The risk of infection is 16% at 10 years and 22% at 20 years. Our patients have an infection rate of 16.1%, with the longest follow-up period of 12 years. Most of the patients with primary and secondary bone tumour had undergone chemotherapy or radiotherapy. The long operating hours in these immunocompromised patients would be among important risk factors for patients to develop infection^16,17^. Periprosthetic infection can be devastating; up to 30% of patients may require amputation after multiple failed attempts to eradicate the infection^13^. In fact, periprosthetic infection was the most common mode of failure in up to 8.3% of cases^8^. The treatment process of periprosthetic infection had impacted negatively on both the treating teams and patients. It required multiple admissions for surgeries and antibiotics administration, which took both time and money. Even if the infection was eventually controlled or treated, the patients’ functional outcome would be significantly reduced^18^. Besides, infection also delayed adjuvant therapy, which could potentially cure the patients and improved their long-term outcome.

Type 5 failure (local recurrence) was noted to have a high proportion of implant failure among our patients, which is usually not reported in other studies. Eight out of 9 patients had Type 5 failure related to osteosarcoma or Ewing’s sarcoma, which had poor chemotherapy responses based on final histopathological reports. In fact, a poor response to chemotherapy with evidence of a low percentage of tumour necrosis signified the aggressiveness of the malignancy and a high tendency of tumour recurrence. Moreover, tumour cells could become cross resistant to a broad spectrum of chemotherapy drugs, which would lead to recurrence and failure of treatment. In another study, 13% of patients eventually underwent amputation, either due to infection or local recurrence^13^. In our series, the amputation rate was 7.1%, which was lower than the reported rate. Undeniably, the incidence of infection might be increased with the prolonged follow-up period as reported in Grimer *et al*, which in turn increased the rate of amputation^13^.

Overall osteosarcoma patients recorded to have the highest number of failures in all types of failure categories with a total of 23 failure (51%) documented for all the failure cases. One of the reasons is patients who sufferred from osteosarcoma accounted for highest number of patients’ population which equalled to 46.4% of all the patients. Wide resection is needed for osteosarcoma associated with extensive soft tissue dissection. The condition would be worse if the tumour was huge, as this had posted additional risk of poor soft tissue coverage. Therefore, the reduced blood supply to the surrounding tissue further exaggerated soft tissue failure (Type 1 failure). On the other hand, high physical demand in young osteosarcoma patients may associated with structural failure (Type 3 failure). The chemotherapy and long operating hours for limb salvage surgery also put patients on higher risk to develop Type 4 failure^16,17^. Eight out of the 9 patients that suffered from Type 5 failure showed poor chemotherapy respond. Poor chemotherapy respond is associated with poor prognosis among patients with osteosarcoma. This group of patients are more likely to suffer from local recurrence and secondary metastasis.

Limitations of this study are the heterogeneity of the patient population and prosthesis locations. Furthermore, one of the limitations of this study was the relatively small samples size with different histological diagnoses. Hence, a more in-depth study could be done in the future with a larger sample size.

## Conclusion

As a summary, the overall result of endoprosthesis reconstructions that were performed in our centre achieved a result that was compatible with other centres around the world. Moreover, it should be performed on a carefully selected patient group to maximise the benefit of the surgery.
